# From Molten Calcium Aluminates through Phase Transitions to Cement Phases

**DOI:** 10.1002/advs.201902209

**Published:** 2019-11-26

**Authors:** Hao Liu, Wenlin Chen, Ruikun Pan, Zhitao Shan, Ang Qiao, James W. E. Drewitt, Louis Hennet, Sandro Jahn, David P. Langstaff, Gregory A. Chass, Haizheng Tao, Yuanzheng Yue, G. Neville Greaves

**Affiliations:** ^1^ State Key Laboratory of Silicate Materials for Architectures Wuhan University of Technology Wuhan 430070 China; ^2^ Department of Chemistry and Bioscience Aalborg University DK‐9220 Aalborg Denmark; ^3^ Department of Physics Aberystwyth University Penglais Campus Aberystwyth Ceredigion SY23 3BZ UK; ^4^ School of Materials Science and Engineering Hubei University Wuhan 430062 China; ^5^ School of Earth Sciences University of Bristol Wills Memorial Building Bristol BS8 1RJ UK; ^6^ Conditions Extrêmes et Matériaux: Haute Température et Irradiation University d'Orléans 45071 Orléans cedex 2 France; ^7^ Institute of Geology and Mineralogy University of Cologne 50674 Cologne Germany; ^8^ School of Biological and Chemical Sciences Queen Mary University of London London E1 4NS UK; ^9^ Department of Chemistry The University of Hong Kong Hong Kong China; ^10^ Department of Chemistry McMaster University Hamilton Ontario L8S 4M1 Canada; ^11^ Department of Chemistry La Sapienza University of Rome Piazzale Aldo Moro 00185 Roma Italy; ^12^ School of Materials Science and Engineering Qilu University of Technology Jinan 250353 China; ^13^ Department of Materials Science and Metallurgy University of Cambridge Cambridge CB3 0FS UK

**Keywords:** aerodynamic levitation, calcium aluminates, cement, fragile–strong phase transitions, molecular dynamic simulation

## Abstract

Crystalline calcium aluminates are a critical setting agent in cement. To date, few have explored the microscopic and dynamic mechanism of the transitions from molten aluminate liquids, through the supercooled state to glassy and crystalline phases, during cement clinker production. Herein, the first in situ measurements of viscosity and density are reported across all the principal molten phases, relevant to their eventual crystalline structures. Bulk atomistic computer simulations confirm that thermophysical properties scale with the evolution of network substructures interpenetrating melts on the nanoscale. It is demonstrated that the glass transition temperature (*T*_g_) follows the eutectic profile of the liquidus temperature (*T*_m_), coinciding with the melting zone in cement production. The viscosity has been uniquely charted over 14 decades for each calcium‐aluminate phase, projecting and justifying the different temperature zones used in cement manufacture. The fragile–strong phase transitions are revealed across all supercooled phases coinciding with heterogeneous nucleation close to 1.2*T*_g_, where sintering and quenching occur in industrial‐scale cement processing.

## Graphical abstract

The evolution mechanism of different calcium‐aluminate phases during fast heating to about 3200 K and subsequent cooling is revealed by measuring their associated thermophysical properties (e.g., viscosity and density) using the self‐developed aerodynamic levitation technique and by bulk‐scale computationally modeling these properties and the atomistic structural changes. The fragile‐to‐strong phase transitions are discovered across all supercooled calcium‐aluminate liquids.

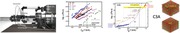

## 1 Introduction

Crystalline calcium‐aluminate phases are the critical setting agents in cement technology.^1^, ^2^, ^3^ The melts from which they crystallize comprise a remarkable glass‐forming system whose compositions, structures and thermophysical properties have not yet been correlated with real‐world cement processing. In particular, the supercooled phases of calcium aluminates in Portland cements (PCs) are also essential for the formation of other more dominant clinker phases such as calcium silicates like Ca_2_SiO_4_ (C2S) and Ca_3_SiO_5_ (C3S).^3^ By contrast calcium‐aluminate phases are dominant in calcium‐aluminate cements (CAC).^3^

The principal cement phases nucleate eutectically during melting and quenching, ranging from CaAl_2_O_4_ (CA) and CaAl_4_O_7_ (CA2) in calcium‐aluminate cements^2^ to Ca_3_Al_2_O_6_ (C3A) in Portland cement (PC),^3^ with Ca_12_Al_14_O_33_ (C12A7) effectively acting as a systemic flux. Individual crystalline structures are well established^2^, ^3^ and corresponding melt and glass structures have been modeled from diffraction and spectroscopy.^4^, ^5^, ^6^, ^7^, ^8^, ^9^, ^10^, ^11^

The calcium‐aluminate glass‐forming system is very extensive compared, for instance, to binary silicates,^12^ incorporating additional oxygen sites to the well‐known bridging O^2^ and nonbridging O^1^ varieties. These include O^3^ triclusters as well as isolated O^0^ species.^4^, ^6^, ^8^, ^9^, ^10^, ^11^ Moreover, not all aluminum polyhedra are tetrahedral Al^IV^, wherein Al^V^ and some more constrained Al^VI^ sites occur;^5^, ^7^, ^9^, ^10^ with occurrent calcium octahedra rarely undistorted.^5^, ^6^ O, Al, and Ca speciation evolves similarly with the composition across calcium‐aluminate liquids and crystalline phases.

The viscosities η and densities ρ of these important cement phases, however, are mostly unknown, yet the relationship between thermophysical and structural properties of supercooled liquids lies at the heart of nucleation and vitrification processes. This relation is well studied for the following two extreme scenarios: one in respect to the liquids with exceptional lability‐like elemental metals,^13^ the other concerning glass‐forming liquids such as silicates^12^, ^14^ where crystallization is inhibited by high η(*T*_m_) values. However, poor glass formers fall between these extremes, with substantial crystal growth rates observed between *T*_m_ and the glass transition *T*_g_.^15^

Calcium aluminates, therefore, demand innovative approaches to understanding glass forming ability (GFA)^16^, ^17^ and the alternative of lability.^15^ For most liquid oxides at supercooled temperatures, η is approximately Arrhenian around *T*_m_, becoming less‐so toward *T*_g_, characterized by the fragility index $m\;={\left(\frac{d(\log\;\eta )}{d(T_{g}/T)}\right)}_{T=T_{g}}\;$.^12^, ^18^ For typical fragile liquids, *m* > 40 while for archetypal glass formers like SiO_2_, the viscosity is Arrhenian throughout the supercooled region, with *m* ≈ 20 constituting *strong* behavior.^12^ As 1.2*T*_g_ is approached, however, the ergodicity of the liquid is lost^12^, ^19^, ^20^, ^21^ and a “dynamic crossover” in structural heterogeneity occurs.^22^, ^23^ This demarcation between fragile and strong glass‐forming liquids has been broken with the discovery of fragile‐to‐strong (*f*‐*s*) transitions in the supercooled region for individual liquids like water,^24^, ^25^ network liquids^26^, ^27^ as well as liquid metals,^28^, ^29^ for which GFA is generally poor.

To date, none have explored the detailed microscopic and dynamic mechanisms of the transition from aluminate liquids, through the supercooled state to glassy and crystalline phases during cement clinker production. The exploration was impossible due to the following three principal factors. First, no direct in situ method can quantitatively assess the viscosity, density, structure, and thermal expansion coefficients from the melting point to the glass transition, due to the intervention of strong crystallization. Second, no simple means can determine structure and properties of equilibrium aluminate liquids, due to the extremely high temperatures involved (2000–3100 °C). Third, it is difficult to vitrify calcium aluminates due to their poor glass forming ability (GFA).

**Figure 1:**
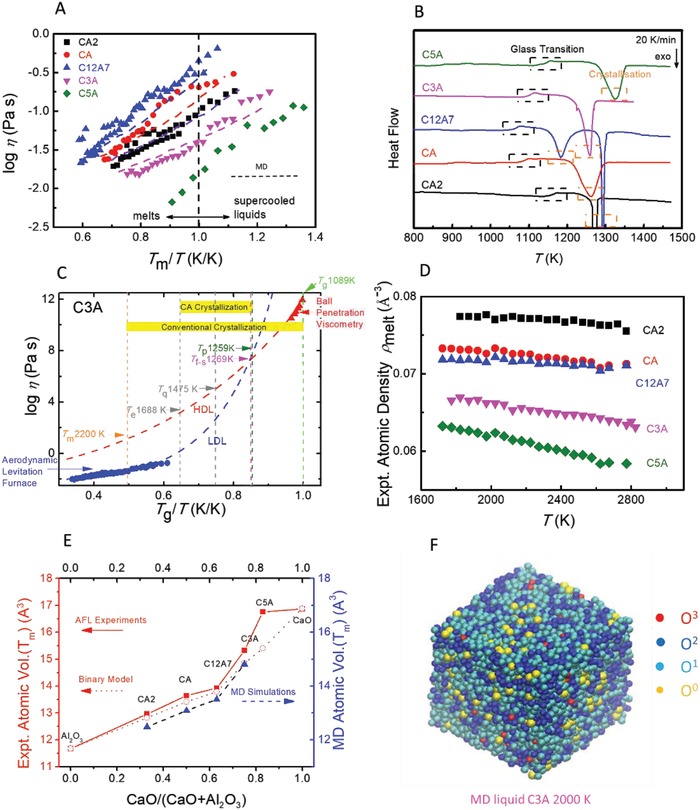
Viscosity, DSC, density, and modeling of calcium‐aluminate cementitious melts. A) logη versus *T*_m_/*T*. Error of viscosity data: <8%. B) DSC determining *T*_g_ and *T*_p_ with an error range of ±2 K. C) logη versus *T*_g_/*T* identifying polyamorphic mismatch and crystallization zone. Error of viscosity data: <10%. LDL: low density liquid (fragile); HDL: high density liquid (strong). D) ρ versus *T*. Error of density: <5%. E) Atomic volume *V*(*T*_m_): experiments versus modeling. F) MD simulation of C3A highlighting oxygen species.

**Figure 2:**
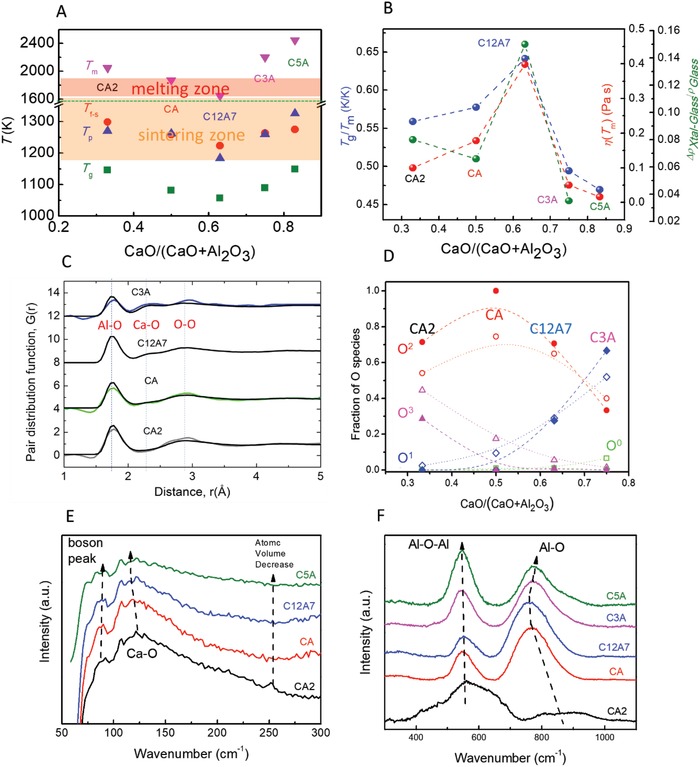
Melting, solidification, and dynamics of calcium‐aluminate cement phases. A) *T*_f‐s_, *T*_p_, and *T*_g_ (HDA) following *T*_m_ eutectic, including cement processing zones and quenching. B) GFA metrics *T*_g_/*T*_m_, η(*T*_m_), and Δρ_cryst–glass_/ρ_glass_. C) Pair distribution functions *G*(*r*) of liquids (colored curves refer to the neutron diffraction results) cf. MD simulations (black). D) Oxygen speciation O^0^, O^1^, O^2^, and O^3^ with calcium‐aluminate composition for MD melt (open points) and crystalline phases (solid points). E) Low‐frequency and F) high‐frequency Raman spectra, showing compositional trends.

**Figure 3:**
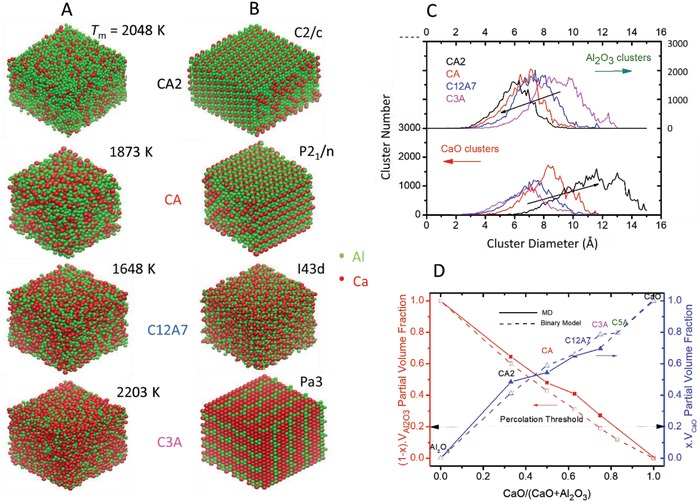
CaO and Al_2_O_3_ substructures in liquid and crystalline calcium‐aluminate phases. A) Visualization of CaO modifier (red) and Al_2_O_3_ network (green) polyhedra in MD melts and B) in crystalline cement phases. C) cluster sizes and numbers for CaO and Al_2_O_3_ substructures in calcium‐aluminate melts. D) Volume fractions *V*(*x*)_CaO_ and *V*(1 − *x*)_Al2O3_ from MD relative to those from a Binary model, where *x* = CaO/(CaO + Al_2_O_3_).

**Figure 4:**
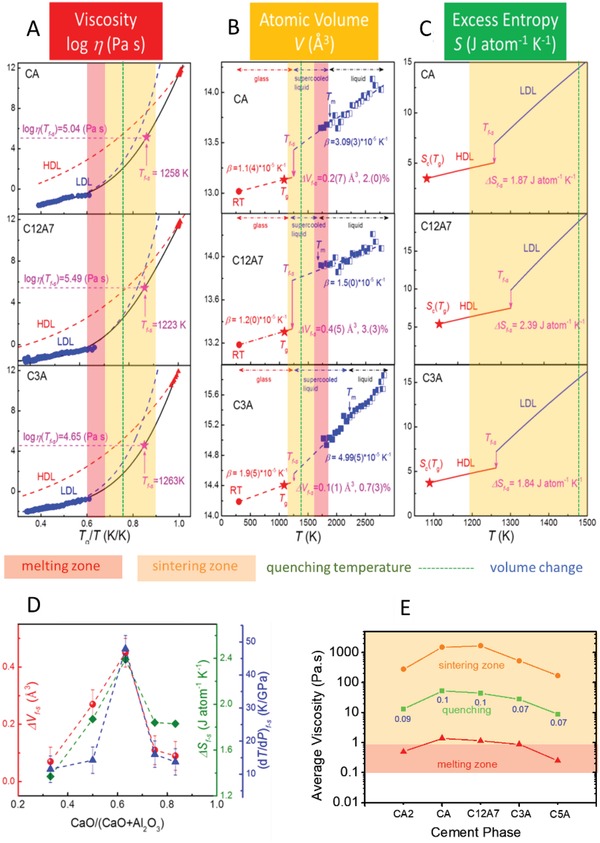
Fragile‐to‐strong transitions and cement processing zones: A) *f*‐*s* transitions identified from η mismatch between liquids around *T*_m_ and *T*_g_, analyzed using the MYEGA equation,^28^ identifying *T*_f‐s_, η(*T*_f‐s_), and LDL and HDL liquid cement phases for CA, C_12_A_7_, and C_3_A. B) Volume steps Δ*V*_f‐s_. C) Entropy steps Δ*S*_f‐s_ at *T*_f‐s_. D) Δ*V*_f‐s_ and (d*T*/d*P*)_f_ *_‐_*_s_ for the calcium‐aluminate system. E) Average η(*T*_melting_), η(*T*_quenching_), and η(*T*_sintering_) for PC furnace zones across calcium‐aluminate phases. Shrinkage on quenching is shown by the values (in blue) beneath the quenching viscosity curve.

Herein, we describe the aerodynamic levitation of laser‐heated calcium‐aluminate samples, to resolve distinct and manifold molten phases and their associated thermophysical properties, embracing industrially relevant temperature ranges of 1450–2200 °C. Complementary bulk‐scale computationally modeling provides supporting resolution of atomistic structural details. For the first time, we report here in situ measurements of η(*T*), ρ(*T*) and coefficients of thermal expansion (CTE) by generating melts of all crystalline calcium‐aluminate phases occurring in cements, using unique aerodynamic levitation furnace (ALF) techniques^30^, ^31^ (Figure S1, Supporting Information), covering temperatures from the eutectic *T*_e_ to well above *T*_m_ (**Figure** **1**). As ALF melting affords contactless conditions and rapid cooling to inhibit heterogeneous nucleation around *T*_m_, we were able to obtain fast‐quenched glasses across all calcium‐aluminate compositions. On reheating, differential scanning calorimetry (DSC) was used to locate *T*_g_ and heterogeneous crystallization *T*_p_, and in addition ball penetration viscometry (BPV) enabled η(*T*) around *T*_g_ to be measured (Figure 1 and **Figure** **2**). Molecular dynamics (MD) simulations of ≈15 000 atoms, over a 50 ns trajectory, successfully reproduced thermophysical properties η, ρ, and CTE, in addition to complementary neutron diffraction pair distribution functions (PDFs), from which both oxygen and aluminum speciations have been determined (Figures 1 and 2). Atomistic structures have also enabled clustering and percolating CaO and Al_2_O_3_ components to be followed in calcium‐aluminate melts in order to compare with crystalline structures (**Figure** **3**). Finally, by coupling AFL with BPV measurements, *f*‐*s* phase transitions *T*_f‐s_ have been identified (Figure 1; Figure S6, Supporting Information) and lie close to crystallization temperatures *T*_p_ (**Figure** **4**). With η(*T*) analyzed throughout the supercooled region for all molten calcium‐aluminate phases, direct comparisons have been made under industrial cement furnace conditions (Figure 4).

## 2 Results

The viscosities of calcium‐aluminate melts at *T*_m_ obtained from ALF measurements, range from 10^−2^ to 10^0^ Pa—very small with respect to other inorganic glass formers,^12^, ^16^ yet comparable to calcium silicate (CS) phases^5^, ^32^ in PCs.^3^ η(*T*_m_) maximizses for the cement flux composition C12A7 (Figure 1A), coinciding with the eutectic minimum in *T*_m_ 3 and replicated in MD simulations. Likewise, when melt‐quenched glasses are reheated, *T*_g_ and *T*_p_ (Figure 1B) also mimic the eutectic minimum in *T*_m_ (Figure 2A), with heterogeneous crystallization *T*_p_ ≈ 1.2*T*_g_, aligning with the dynamic crossover in supercooling.^18^, ^19^, ^20^, ^21^, ^22^, ^23^ Combining η(*T* > *T*_g_) with η(*T* < *T*_m_) spans up to 14 decades of logη versus *T*_g_/*T*, furnishing a wide thermohistory from which, using recent analysis methods, viscosity can be tracked reliably from *T*_m_ to *T*_g_.^28^ This reveals a mismatch in all calcium‐aluminate phases between fragile and strong character around *T*_f‐s_ that is close to 1.2*T*_g_. The phases illustrated in Figure 1C are identified as low‐ and high‐density liquids, respectively, LDL and HDL.^12^ Direct comparisons can now be made with industrial‐scale rotary kiln cement processing zones (Figure 4E; Figures S6 and S7, Supporting Information).

High temperature atomic density falls with CaO content (Figure 1D), whilst the CTEs of the glass slightly increases with CaO (Figure S3, Supporting Information). The corresponding atomic volume at *T*_m_ rises steeply with increasing CaO; well‐reproduced by MD simulations (Figure 1E). In addition, associated atomic coordinations reveal remarkable diversity in oxygen and aluminium speciation (Figure 1F; Figure S5, Supporting Information).

The calcium‐aluminate eutectic *T*_m_ profile,^2^, ^3^ which is mirrored in *T*_g_ as well as *T*_p_ and *T*_f‐s_ (Figure 2A), provides insight into GFA (Figure 2B), as do the melting temperature viscosity η(*T*_m_) and the Δρ/ρ_glass_ ratio, where Δρ is the crystal–glass density difference (ρ_cryst_ − ρ_glass_). These all share the same compositional profile (Figure 2B), relevantly verifying and quantifying the poor GFA of calcium‐aluminate phases.

Our atomistic MD simulations serve as computational models of experimental liquid thermophysical properties, (Figure 1) whilst also predictively reproducing published neutron pair distribution functions;^6^, ^9^ notably the distinctly differing short‐range ordering of [CaO_6_] octahedra and [AlO*_n_*] polyhedra (Figure 2C). Moreover, the oxygen speciation common to thermophysical and structural properties (Figure 1F; Figure S5, Supporting Information) changes significantly with composition (Figure 2D). The existence of O^3^ triclusters is predicted in CA2. The fraction of triclusters decreases with CaO and disappear in CA,, i.e., CA contains only bridging oxygens O^2^. With further increasing CaO, O^2^ decreases, while nonbridging oxygens (O^1^) increases, and finally there are 32% O^2^ and 68% O^1^ in C3A. Importantly, corresponding crystalline phases also share very similar oxygen speciation (Figure 2D), therefore, linking their local structures closely to their melts.

Raman spectra of glasses display a rise in the THz boson peak intensity expected as the density decreases^33^, ^34^ with increasing CaO, whilst the CaO_6_ rattling frequency decreases from an O^2^ to an O^1^ environment (Figure 2E). The Al—O—Al bending mode, though, shows negligible variation;^7^ the main O^2^ bridging angle remaining at ≈120°, while the optic bond stretching Al—O frequency falls and then rises, pivoting around the eutectic C12A7 (Figure 2F). Increasing network disorder with increasing Ca‐content disperses neighboring THz modes, evidenced in the widening boson peak (Figure 2E).

As local structures of the network modifier (CaO) and network former (Al_2_O_3_) in calcium‐aluminate melts differ greatly (Figure 2C), we have explored whether clustering of these component features in long range order and the degree to which clusters are interconnected, with respect to crystalline structures. The spatial distribution of Ca and Al polyhedra in calcium‐aluminate melts at *T*_m_ (Figure 3A) suggests interpenetrating aperiodic network and modifier substructures developing counter wise with composition. These appear to mimic the development of periodic distributions in crystalline calcium aluminates from the fully polymerized aluminate network (CA2) through the layered (CA) to unpolymerized (C3A) structures (Figure 3B). CaO and Al_2_O_3_ cluster sizes analysed from melt structures at *T*_m_ increase with respective atomic volume fractions *V*_Al2O3_ and *V*_CaO_. This also includes the percolation threshold of ≈0.2 volume fraction for jammed polyhedral spheres (Figure 3C,D; Figure S4, Supporting Information).^35^ This occurs around CA6 for CaO and C3A in Al_2_O_3_, so that above these thresholds, modifier and network clusters in calcium‐aluminiate melts (Figure 3D) are separately interconnected.

The mismatch in fragility between the LDL (high temperatures) and HDL (low temperatures) phases for C3A (Figure 1C) is observed for all supercooled calcium aluminates (Figure 4A; Figure S7, Supporting Information). Analyses^28^ of AFL‐BPV data also provides mean LDL–HDL viscosity values from which fluidity can be predicted during sintering, melting and quenching stages of cement processing (Figure 4E). Using CTEs for melts and glasses (Figure S3, Supporting Information), the atomic volume has been extrapolated from high and low temperatures, identifying steps in Δ*V*_f‐s_, together with complementary parallel entropy steps Δ*S*_f‐s_, obtained by Adam–Gibbs analysis^12^ of the polyamorphic liquid states (Figure 4B,C; Figure S6, Supporting Information). From the Clausius–Clapeyron relation, the slope of the phase boundary between coexistent liquid states d*T*/d*P* = Δ*V*_f‐s_/Δ*S*_f‐s_ is found to be positive for all calcium‐aluminate cement phases (Figure 4D), implying an increased disorder with expansion, affected through the HDL–LDL transition. This is also displayed in the *f*‐*s* transitions observed in metals,^28^, ^29^ but is distinct from less well‐packed oxide polyamorphic networks, where d*T*/d*P* is negative.^26^, ^27^

## 3 Discussion

The ubiquity of *f*‐*s* transitions (Figure 4A) across all supercooled calcium‐aluminate cement phases is significant, in that *T*_f‐s_ values closely follow the profile of the eutectic minimum and, in particular, match heterogeneous crystallization values *T*_p_ (Figure 2A), suggesting that nucleation of calcium‐aluminate phases is promoted by these transitions. As this also coincides with the dynamic crossover at ≈1.2*T*_g_,^21^, ^22^, ^23^, ^24^ the breakdown of the classical reciprocity of viscosity and diffusion, coupled with poor GFA (Figure 2), may also be contributory factors.

GFA is often quantified by *T*_g_/*T*_m_, with good glass formers (like SiO_2_) lying above the 2/3 Kauzmann average,^12^, ^36^ often accompanied by large values of η(*T*_m_), typically ≥10^5^ Pa s;^16^, ^17^ both metrics reflecting foreshortened supercooled ranges. These good GFA metrics also require there to be larger Δρ/ρ_glass_ values compared to poor GFA metrics, effectively reducing the tendency to nucleation. Conversely the same criteria single out calcium‐aluminate phases as poor glass‐formers due to low average *T*_g_/*T*_m_ (0.55), low η(*T*_m_) (≈0.1 Pa s), and small Δρ/ρ_glass_ (≈0.09) (Figure 2B). Given 14 decades of supercooling (Figure 1C), quenched calcium‐aluminate glasses themselves are only obtainable under contactless ALF conditions, but readily recrystallize on heating (Figure 1B). The heterogeneous crystal growth in glass‐forming systems generally starts at ≈*T*_g_ and reaches a maximum at a temperature close to, yet beyond *T*_m_.^15^ For single calcium‐aluminate phases, nucleation commences at ≈1.2*T*_g_ (Figure 2A), whereas, for cement processing, it extends to just below the eutectic minimum at *T*_e_ ≈ 1.5*T*_g_.^2^ Nucleation therefore covers a reduced range of *T*_g_/*T*_m_ ≈ 0.65–0.85 relative to the wider range of ≈0.5–1.0 for the conventional cases (Figure 1C). Additionally, cooperative eutectic growth rates are significantly lower than those for single phase crystallization,^13^, ^15^ and thus the eutectic crystal growth will further reduce the lability, consistent with the formation of only tiny crystals, i.e., the ≈50 µm calcium‐aluminate crystallites observed elsewhere.^2^, ^3^ Given the measured CTEs for calcium‐aluminate melts, glasses and crystals (Figure S3, Supporting Information), the substantial volume decrease of ≈0.09 occurs during quenching of melts toward crystals, and hence effectively expanding voids between the crystalline particles (Figure 4E), which explains the characteristic porosity within CAC and PC;^2^, ^3^ functionally beneficial to the ingress of water during cementation.

Furnace melting zones (Figure 2A) notably just extend from *T*_m_ of the CAC setting phase CA to that of C12A7, the fusion of C3A therefore exploits the metastable kinetics of eutectic melting.^13^ Using the combined ALF‐BPV viscosities (Figure 4A), average melting zone values for calcium aluminates range from η ≈ 0.1 to 1 Pa s, in line with normal melting (Figure 4E). Likewise, average sintering zone values extend from η ≈ 100–1000 Pa s, covering the working points traditionally used in glass technology (Figure 4E). The thermophysical properties of single calcium‐aluminate phases reported here therefore quantify the fluidities at each stage in CAC processing. As η(*T*_m_) for calcium‐silicate phases is comparable to that for calcium‐aluminate phases,^5^, ^32^ similar outcomes will also apply to PC processing.

Figure 4A also demonstrates that *f*‐*s* transitions fall toward the bottom of the sintering zone and involve significant shrinkage (Δ*V*_f‐s_) and release of energy (*T*_f‐s_·Δ*V*_f‐s_; Figure 4B,C). Moreover, PC melt quenching temperature *T*_q_ is typically midway between *T*_m_ and *T*_p_ (Figure 1C) within the cement‐sintering zone (Figure 4). Predicted viscosities (Figure 4A) lie close to the practical melt‐homogenization viscosity (≈1 Pa s) during glass production. If quenching, however, is most likely to result in crystallization dictated by *f*‐*s* transitions at the dynamic crossover temperature of ≈1.2*T*_g_, there may be scope from quenching temperatures to the eutectic *T*_e_ where the melt might be more homogenous and more fluid. Additional heat would be released on cooling, which could augment furnace preheated combustion air.

The binary model included in Figures 1E and 3D (Figure S2, Supporting Information) closely follows the experimental and MD determined atomic volumes of the melts, demonstrating that the partial atomic volumes of the percolating modifier and network substructures lie close to those of the respective end‐members: CaO and Al_2_O_3_ (Figure S2, Supporting Information). Given that calcium‐aluminate melts and crystals share very similar oxygen speciation (Figure 2D; Figure S5, Supporting Information), this will assist liquid–solid transitions in cement processing (Figure 4), with local order for particular melt compositions fluctuating around corresponding crystal structures (Figure 3A,B), in turn promoting nucleation and subsequent growth. As CaO and Al_2_O_3_ substructures in crystalline calcium aluminates are intimately configured despite their respective ionic and directional bonding being quite different, it is reasonable to assume that hydration reactions might initially progress sequentially. This is the outcome of recent C3A force field calculations,^37^ which have shown Al_6_O_18_ rings first hydrating and then fragmenting into octahedral complexes. This reconfiguration of sixfold aluminate rings, consistent with ^27^Al‐NMR observations during cement setting,^38^ is accompanied by agglomerating amorphous layers of Ca(OH)_2_.^37^

Turning finally to solid–liquid transitions in cement processing, given that the end members are highly refractory, namely, Al_2_O_3_ (*T*_m_ 2293 K) and CaO (*T*_m_ 2843 K), and that melting relates to a dramatic reduction in bulk modulus, the minimum in *T*_m_ around midrange composition (Figure 2A) suggests that the rigidity of each component is progressively compromised as its share of atomic volume decreases. The observed changes in oxygen speciation (Figure 2D) demonstrate how the prevalence of O^2^ links in the β‐tridymite fully polymerized network of CA endows more structural integrity than the O^1^ and O^2^ mix of depolymerized Al_6_O_18_ rings in C3A. Conversely, Ca environments become increasingly irregular as CaO concentration decreases, from four perfect octahedral sites and two octahedral sites in C3A, to distorted sixfold and ninefold sites in CA.^5^ Oxygen, calcium and also aluminum environments generally transfer to corresponding melt structures.^6^, ^38^ Current thinking on melting relates the crystal–liquid transition to incipient rare events culminating in the occurrence of instantaneous yet property‐driving nanostructures at *T*_m_.^39^ For Ca cement phases, η(*T*_m_) values reported here are some of the smallest recorded (Figure 1A; Figure S3, Supporting Information) and the supercooled ranges of these poor glass formers are huge (Figure 1C and 4D), favoring the reoccurrence of localized melting with reduced steric hindrance. As such, the role of collective long wavelength, anharmonic THz vibrations comprising the boson peak (Figure 2E) will dominate the Δ*S*_f‐s_ contribution to LDL. If ps periods were on the time scale of the occurrence of rare events in CA phases, then these modes would appear to be important in cooperatively triggering melting as previously found in zeolites.^40^

## 4 Conclusions

The relatively lower ratios of the glass transition temperatures (*T*_g_) to the melting temperatures *T*_m_ for calcium‐aluminate (CA) phases indicate that these phases are poor glass formers, thus determining the crystalline nature of the aluminate phases in cement. Fragile‐to‐strong phase transitions are ubiquitous across all supercooled calcium‐aluminate phases. Moreover, they coincide with the heterogeneous crystallization observed at ≈1.2*T*_g_—exactly where sintering and quenching occurs in large‐scale industrial cement manufacture. Viscosities of all calcium‐aluminate phases have been uniquely determined, covering 14 decades, and projected onto the different temperature zones in cement manufacture.

Molten calcium‐aluminate phases comprise interpenetrating CaO and Al_2_O_3_ phases with atomic speciation closely following crystalline structures. This explains the facile nucleation in the processing of cement, crystallite shrinkage in the polycrystalline matrix, and the initial hydration reactions.

## 5 Experimental Section

*Synthesis*: In the *x*CaO–(1 − *x*)Al_2_O_3_ system, C5A (*x* = 0.83), C3A (*x* = 0.75), C12A7 (*x* = 0.63), CA (*x* = 0.5), and CA2 (*x* = 0.33) glass spheres were synthesized by liquid‐quenching CaO–Al_2_O_3_ precursors in the aerodynamic levitator furnace. The precursors were prepared by a sol–gel method,^41^ using Al(OBu)_3_ 97% purity and (Ca(NO_3_)_2_·4H_2_O) 99% purity. Auxiliary materials were Etac 98% purity, hydrochloric acid with a concentration of 38%, and ethanol with purity of ≥98%. With a mole ratio of 1:1, Al(OBu)_3_ and Etac were magnetically stirred for 30 min (600 rpm) and then mixed ultrasonically for 1 h to form a stable transparent mixture. This was diluted in ethanol, deionized water, and hydrochloric acid, with a mole ratio of 1:4:3:0.15, and magnetically stirred at room temperature (RT) for 24 h (600 rpm) to form the first solution. The second solution was prepared using Ca(NO_3_)_2_·4H_2_O dissolved in ethanol with extra deionized water (Ca(NO_3_)_2_·4H_2_O:H_2_O = 3:4) to launch partial hydrolysis using an ultrasonic bath (1000 Hz) for 30 min followed by magnetic stirring at RT for 24 h (600 rpm). Based on the stoichiometric composition of *x*CaO–(1 − *x*)Al_2_O_3_, the two solutions were mixed and magnetically stirred at RT for 24 h (600 rpm) allowing the formation of homogenous CaO–Al_2_O_3_ solutions. After drying at RT for 3 days and heat‐treating at 600 °C for 3 h, CaO–Al_2_O_3_ powders formed.

*Aerodynamic Levitation Furnace and Viscosity Measurements*: The ALF is based around an aerodynamic conical converging–diverging levitator nozzle with a central opening and depth chosen to accommodate a 2 mm sphere as described in refs. 30, 31. Levitation gas passed through an acoustic stimulation chamber using a programmable frequency generator before entering the nozzle (Figure S1, Supporting Information). The sample sphere geometry was determined using a shadow casting laser in conjunction with a high‐speed camera operating at up to 4000 fps using a telecentric lens to minimize perspective effects caused by sample movement. A narrow pass filter at the laser wavelength eliminated self‐illumination from the incandescent sample. Sample heating was provided by 80 and 40 W lasers heating the top and bottom of the sample, respectively. To precisely monitor sample temperature, fiber coupled single and dual color pyrometers were used with a software control loop to control the laser power, stabilizing the temperature to within ±5 °C, Figure S1 (Supporting Information) shows the ALF layout and image sequence of the liquid drop with time. In addition to melting and levitating liquid spheres, glasses for characterizations were formed by quenching at about 1000 K s^−1^ by switching off the lasers.

Using a conventional sine wave to acoustically oscillate the levitated liquid drop vertically

(1) $$r_{h/v}\;\left(t\right)=\;A\cdot \sin\left[2\pi v_{\mathrm{ho}}\left(t-t_{0}\right)\right]+r_{0}$$

where *r*_h_(*t*) and *r*_v_(*t*) are the horizontal and vertical drop radius (average *r*_0_), *A* is the amplitude, *v*_ho_ the excitation frequency, and *t*_0_ is an offset. For the damped decay after the gas‐flow oscillator has been switched off

(2) $$r_{h/v}\;\left(t\right)=\;A\cdot \exp\left[-\Gamma \left(t-t_{1}\right)\right]\cdot \sin\left[2\pi v_{\mathrm{dho}}\left(t-t_{0}\right)\right]+R_{\mathrm{av}}$$

where Γ is the damping constant of the oscillation, *t*_1_ is the starting time, *v*_dho_ the resonance frequency of the drop, and *R*_av_ the drop radius at rest. The time dependence of the drop radius (*R*) is associated with Γ (Figure S1, Supporting Information), from which the viscosity η can be calculated^30^, ^31^

(3) $$\eta \;=\frac{1}{\left(l-1\right)\left(2l+1\right)}\;\;\frac{3M\Gamma }{4\pi R}=\frac{\Gamma \rho R^{2}}{5}$$

where *l* is the oscillation mode, the primary resonance frequency being dominated by *l* = 2 and order *m* = 0.^30^, ^31^ In Equation (3), *M* and ρ are the drop mass and the density, respectively.

While the viscosities of CaO–Al_2_O_3_ liquids were determined by ALF as described above, the viscosity of C3A bulk glass in the glass transition region was measured using a ball penetration viscometer (BÄHR, VIS405).^42^ The C3A bulk glass was cut to dimensions of approximately 10 × 10 × 5 mm^3^ with the top and the bottom surfaces polished parallel. The viscosities of other CaO–Al_2_O_3_ glasses were estimated by using the relation of viscosity near *T*_g_ and cooling rate *q*_c_

(4) $$\log\eta \left(T_{f}\right)=\log K_{c}\;-\log q_{c}\left(T_{f}\right)$$

where log *K*_c_ is the shift factor, *q*_c_ is the cooling rate (K s^−1^), and *T*_f_ is the fictive temperature where the structure of the equilibrium liquid is frozen‐in and is proportional to the cooling rate.^43^, ^44^ Here, *T*_f_ (Figure 1B) is determined as the onset temperature of the glass transition in DSC upscan curves at different scanning rates (5–30 K min^−1^). For the silicate glasses, log *K*_c_ = 11.35.^44^ Here, log *K*_c_ = 11 is determined for CaO–Al_2_O_3_ glasses by combining of DSC data with viscosity data of C3A bulk glass. Using Equation (4), the viscosity near *T*_g_ for other CaO–Al_2_O_3_ glasses (Figure 1C and 4A) were estimated from their DSC data.

*Thermophysical Properties Measurements*: Crystal–glass density deficit Δρ_cryst–glass_/ρ_glass_ values shown in Figure 2D were obtained from the RT densities of the crystals and glasses. Experimental glass densities were determined using the Archimedes principle with ethanol as the immersion liquid, each sample being measured ten times. The maximum glass density coincided with the eutectic composition, the glass atomic volume increasing monotonically with increasing CaO content (Figure S2, Supporting Information).

The temperature dependence of the liquid densities was obtained from the liquid volume determined from ALF images and the mass from rapidly quenched glass drops (Figure 1E). For the volume coefficients β of thermal expansion (CTE), the rotational symmetry of the ellipse around the vertical axis was assumed. Mean linear CTEs α (=β/3) for CaO–Al_2_O_3_ liquids covered the extended temperature range 1700–2800 K and included the melting points *T*_m_ of the various crystalline phases (Figure S3, Supporting Information).

*Structure Measurements*: The neutron diffraction measurements (Figure 2C) were obtained for CaO–Al_2_O_3_ liquids using an ALF with a vanadium nozzle installed on the D4C diffractometer^45^, ^46^ at the Institut Laue–Langevin. Experimental details and analysis are given in refs. 6, 9, 47.

Raman spectroscopy measurements (55–1600 cm^−1^) on liquid‐quenched glasses were performed at RT using a Renishaw inVia micro‐Raman spectrometer (532 nm). Measurements were performed three to five times on various surface locations for each glass sample, exhibiting no detectable deviation, indicating that the glasses were macroscopically homogeneous for each composition. Spectra (Figure 2E,F) were baseline‐corrected and normalized to a common area.

*Calorimetric Measurement*: The calorimetric measurements were performed using a simultaneous thermal analyzer (STA 449 F1, Netzsch, Selb, Germany), combining differential scanning calorimetry (DSC) with thermogravimetry (TG) in a purged argon atmosphere (40 mL min^−1^). The heat flow of the DSC second upscan for liquid‐quenched specimens (Figure 1B) was used to obtain the enthalpic response, with a prior cooling rate of 20 K min^−1^. The glass transition temperature *T*_g_ was determined by the onset temperature of the endothermic step with an upscan rate of 20 K min^−1^.

*Computer Simulations*: DLPOLY classic^48^ was used for molecular dynamics (MD) simulations, the interatomic potential model constructed by reference to that used to model the structure of calcium‐aluminate crystals. Parameters for the Buckingham Potential $\phi_{12}\;(r)=\;A\cdot \exp\left(-\frac{r}{B}\right)-\frac{C}{r^{6}}$ are shown in Table S1 (Supporting Information). Well‐defined crystal structures from ICDD PDF‐4 and AMCSD databases for CA2, CA, C12A7, and C3A were used as starting points, creating 20 160, 10 080, 14 750, and 16 896 atom ensembles, respectively. Simulations were performed at constant pressure/temperature (NPT), temperature being controlled using Hoover thermostats with a relaxation time of 1 ps. Constant pressure was maintained by applying isotropic barostats with the same relaxation time. Initial heating runs of 500 000 production steps with 200 000 equilibration steps were performed using a time step of 1 fs in order to obtain a molten structure at 4000 K. 12 runs of 50 000 steps, with a decrease of temperature by 100 K between each, were then performed to reach the experimental viscosity temperature range 1700–2800 K, followed by a further 25 steps for annealing to RT, giving an overall cooling rate of 1 K ps^−1^. Melt viscosities (Figure 1A) were obtained from diffusivities *D* inverted to η via the Eyring equation as described in ref. 49.

Modifier and network cluster sizes within melt structures (Figure 3A) were obtained by removing first CaO and then Al_2_O_3_ components from each simulated structure and analyzing respective pore sizes and volumes *V*(*x*)_CaO_ and *V*(1 − *x*)_Al2O3_ (Figure 3C; Figure S3, Supporting Information) using zeo++^50^ (Figure S5, Supporting Information).

## Conflict of Interest

The authors declare no conflict of interest.

## Supporting information

Supporting Information
