# Supplementary material for: From Molten Calcium Aluminates through Phase Transitions to Cement Phases
Source: Adv Sci (Weinh). 2019 Nov 26;7(2):1902209. doi: 10.1002/advs.201902209 (PMC6974954; doi:10.1002/advs.201902209)
Supplement: Supplementary file 1 — Supporting Information [file ADVS-7-1902209-s001.pdf]

Copyright WILEY-VCH Verlag GmbH & Co. KGaA, 69469 Weinheim, Germany, 2019.

ADVANCED SCIENCE
Open Access

Supporting Information

for *Adv. Sci.,* DOI: 10.1002/advs.201902209

From Molten Calcium Aluminates through Phase Transitions to Cement Phases

Hao Liu, Wenlin Chen, Ruikun Pan, Zhitao Shan, Ang Qiao, James W. E. Drewitt,
Louis Hennet, Sandro Jahn, David P. Langstaff, Gregory A. Chass, Haizheng Tao, Yuanzheng Yue, and G. Neville Greaves**

Copyright WILEY-VCH Verlag GmbH & Co. KGaA, 69469 Weinheim, Germany, 2019.

Supporting Information

From Molten Calcium Aluminates through Phase Transitions to Cement Phases

Hao Liu, Wenlin Chen, Ruikun Pan, Zhitao Shan, Ang Qiao, James W. E. Drewitt, Louis Hennet, Sandro Jahn, David P. Langstaff, Gregory A. Chass, Haizheng Tao, Yuanzheng Yue, and G. Neville Greaves**

1. Supplementary Method 1. Aerodynamic Levitation Furnace and Modeling

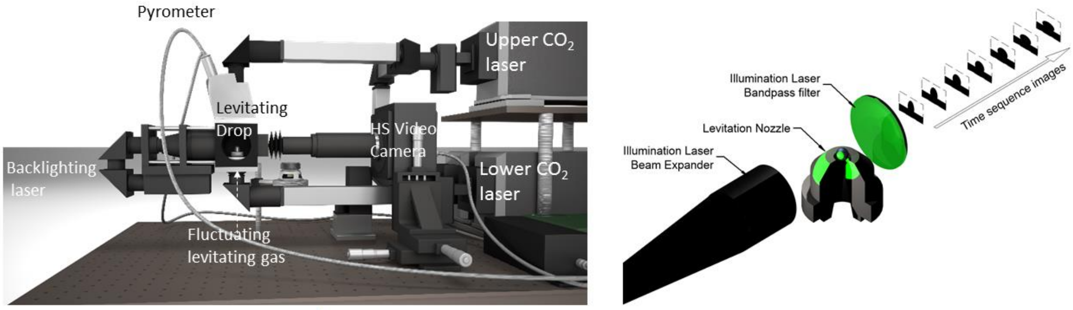

Figure S1. Layout of ALF (top left), Back lighting and bandpass filter with time sequence images (top right). Details methods and in ref 30 and 31.

2. Supplementary Method 2. Calcium Aluminate Glass and Liquid Atomic Volumes and the Binary Model

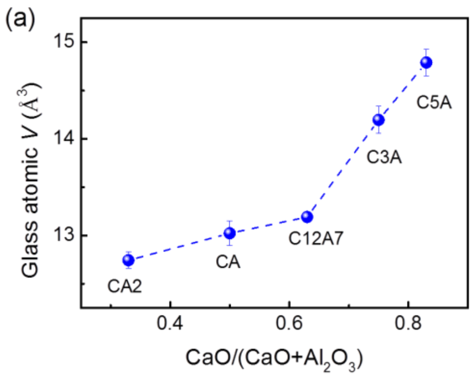

Note: This paper is dedicated to Professor G. Neville Greaves life's work in physics and materials science. The advances made herein embody his seminal contributions to the understanding of the structure and properties of glasses and liquids, as well as his vision for novel experimental and theoretical approaches.

Figure S2. Variation of glass atomic volume with CaO/(CaO+Al_2O_3) ratio measured using Archimedes' principle. It has a similar profile to liquid atomic volume at T_m $V(T_m)$ plotted in Figure 1E, which is replicated quantitatively by MD simulations.

It is also closely followed by a Binary Model for atomic volume simply defined by

$$V(T_m) = (1-x)V_{Al2O3}(T_m) + x\, V_{CaO}(T_m)$$

where x is CaO/(CaO+Al_2O_3), and $V_{Al2O3}(T_m)$ and $V_{CaO}(T_m)$ are the atomic volumes at CA melting points T_m for the end members of the calcium aluminate series, viz. liquid Al_2O_3 and liquid CaO respectively. The Binary Model was introduced to establish in a simple way how the demarcation between Al_2O_3 and CaO subcomponents that characterizes the crystalline phases (Figure 3B) is also present in the liquids from which they nucleate. This is not visually obvious from simulated structures (Figure 3A).

The atomic volume of liquid Al_2O_3 at its melting point V_{Al2O3}(2345 K) was taken from experiment[30] but for liquid CaO V_{CaO}(2845 K) has not yet been measured. We therefore took the RT atomic volume of CaO and published measurements of CTE at high temperatures[51,52] and extrapolated to T_m=2845 K. The crystalline volume for CaO at the melting point was then increased by 5% to allow for the melting volume change, a value typical for close packed crystals,[53] in order to obtain V_{CaO}(2845 K). To predict $V_{Al2O3}(T_m)$ and $V_{CaO}(T_m)$ at the melting points of the CA2, CA, C12A7 and C3A and directly compare with ALF experiments (Figure 1E), experimental coefficients of thermal expansion (CTEs) for Al_2O_3 [30] and CaO[51] were used.

The Binary Model is also in close agreement with partial atomic volume fractions obtained from cluster analysis of MD simulations (Figure 3D), described below. The partial atomic volumes fractions plotted in Figure 3D are defined by $V(x)_{Al2O3} = (1-x) \cdot V_{Al2O3}(T_m)/V(T_m)$ and $V(x)_{CaO} = x \cdot V_{CaO}(T_m)/$ $V(x)_{Al2O3} = (1-x) \cdot V_{Al2O3}(T_m)/V(T_m)$ where $V(x)_{Al2O3}$ and $V(x)_{CaO}$ are the ALF experimental atomic volumes at the T_m of the CA phases.

$V(x)_{Al2O3}$ and $V(x)_{CaO}$ scale almost linearly with CaO content x=Ca)/(CaO+Al_2O_3) indicating that the densities of the Al_2O_3 and CaO subcomponents for the different compositions of liquid CA phases are virtually unchanged, equating with the those of the end members.

Partial atomic volume fractions also enabled the compositions of the percolation thresholds for each subcomponent to be located in liquid CA phases, using the threshold for polyhedral sphere.[44] Between CaO and CA6, CaO clusters are connected but between CA6 and Al_2O_3 they are not, and between Al_2O_3 and C3A Al_2O_3 clusters are connected, yet between C3A and CaO they are not.

3. Supplementary Method 3. Liquid, Glass and Crystal Coefficients of Thermal Expansion (CTE)

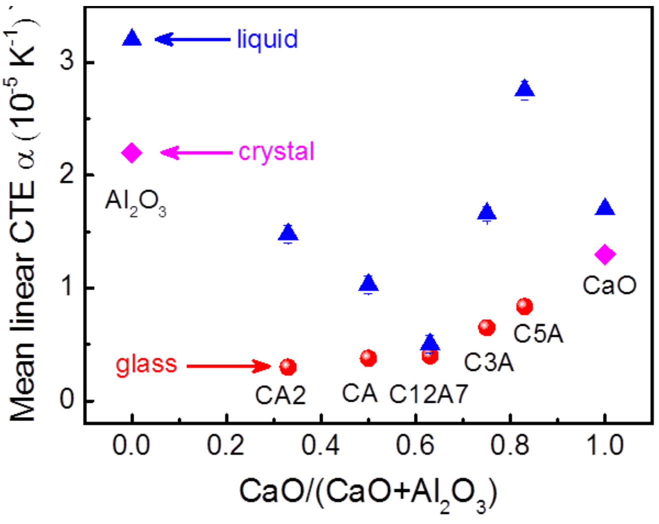

Figure S3. Mean linear coefficients of thermal expansion (CTE) α for glasses (red spheres) and for crystals (blue triangles) taken from refs. 51-54.

Linear Coefficients of Thermal Expansion (CTEs) for CA glasses are extremely small ~3 to $9{\cdot}10^{-6}$ K^{-1}, comparable to heat resistant borosilicate glasses such as Pyrex. For liquids α is ~x3 larger but exhibits a sharp minimum at the eutectic C12A7 composition, suggesting the

Morse potential is very shallow at this composition but deepens either side. The initial decrease (CA2 to C12A7) coincides with a decline in $Al^{V,VI}$ species and oxygen triclusters (Figure 2D) and the formation of a Continuous Random Network.[12] Thereafter (C12A7 to CA5), the AlO_4 network breaks down forming a network deficient liquid[12] with an increase in CTE, coinciding with an increase in Al^{IV} configurations and in NBO's (O^1), together with a decrease in BO's (O^2) (Figure 2D); all trends pointing to an overall increase in network disorder.

Liquid CTEs were used in S2 to obtain atomic volumes the Binary Model with the melting temperatures of CA phases. Liquid and glassy CTEs were also used to define the volume step at the *f*-*s* transitions at $T_{f\text{-}s}$ in Figure 4B and Figure S6.

4. Supplementary Method 4. MD Simulations and Cluster Analysis

Element 1	Element 2	A/eV	B/Å	C/eV Å^6
O	O	1844.0000	0.343645	192.58
Al	O	12201.417	0.195628	31.997
Ca	O	7747.0000	0.252600	93.10

Table S1. Parameters for the Buckingham Potential, with values for A, B and C constants for each atom-pair

MD simulations using DLPOLY classic[35] are described in Methods. The parameters for Buckingham Potential are given in tab. S1. Starting from the multiple unit cell crystalline ensembles – 20160 (CA2), 10080 (CA), 14750 (C12A7) and 16896 (C3A) atom ensembles – illustrated in Figure 3B, these were melted at 4000 K and the cooled to 1700-2800 K, where atomic diffusivities D_i were calculated and inverted to η via the Eyring equation as described in ref. 36.

Liquid CA structures are contrasted with crystalline structures in Figure 3 which shows the Ca (red) and Al (green) positions, each represented by 2.4 Å and 1.75 Å spheres, their respective polyhedral radii. CaO and Al_2O_3 cluster sizes within the melts were obtained by respectively removing Ca and then Al polyhedral and analyzing the resultant pore sizes and volumes $V(x)_{CaO}$ and $V(1\text{-}x)_{Al2O3}$ left behind, using the porous material analysis package zeo++.[37]

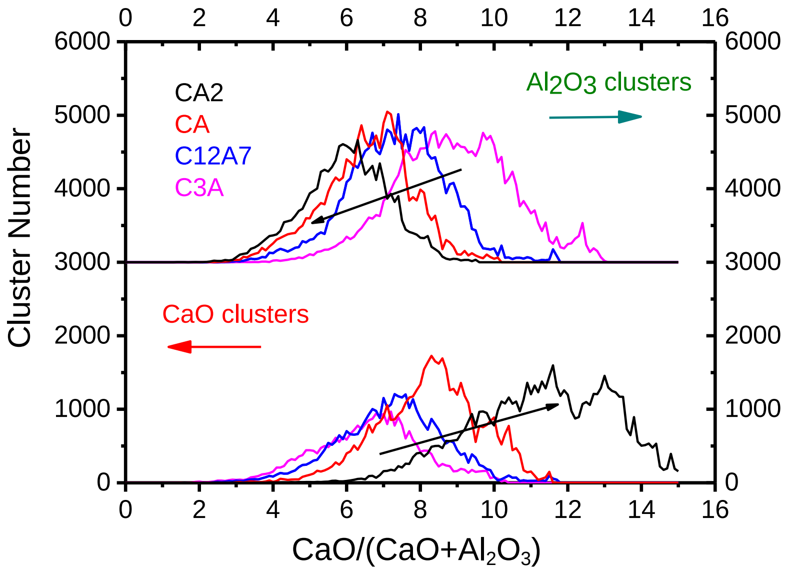

Figure S4A. CaO and Al_2O_3 cluster distributions for MD generated molten CA phases, showing increase in CaO cluster radii with increased CaO/(CaO+Al_2O_3) and corresponding decrease in Al_2O_3 cluster radii.

Following integration these distributions yielded the partial atomic volume fractions $V(x)_{CaO}/V(T_m)$ and $V(1\text{-}x)_{Al2O}/V(T_m)$ for CA2, CA, C12A7 and C3A plotted in Figure 3D, where $V(T_m)$ is the corresponding ensemble box size. We draw attention to the inherent uncertainty in measuring internal volumes absolutely using zeo++ pointed out by Willems et al[40] which relates to the radius of the probe sphere used to measure internal volume in porous structures. This is a fractal problem but, in our case, as

$$V(x)_{CaO}/V(T_m) + V(1\text{-}x)_{Al2O}/V(T_m)=1$$

We were able to trial different probe radii (Figure S4B), which demonstrated that 0.1 Å was appropriate for these systems.

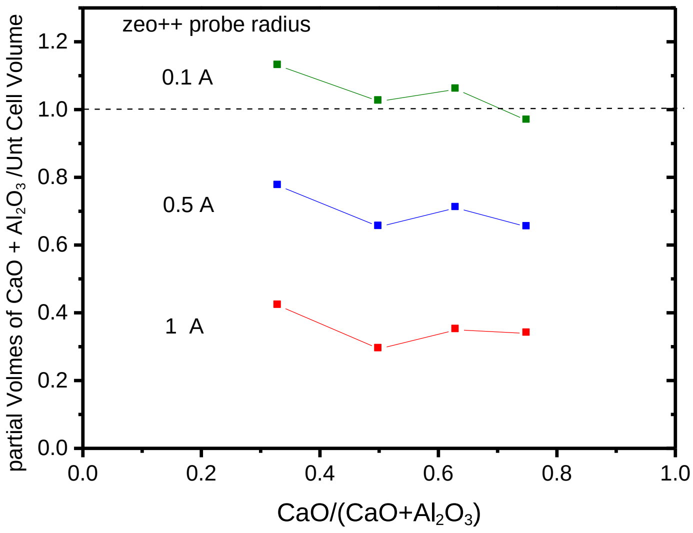

Figure S4B. Summation of atomic volume fractions of CaO and Al_2O_3 $V(\mathrm{x})_{CaO}/V(T_m)$ + $V(1\text{-}\mathrm{x})_{Al2O}/V(T_m)$ for probe radii 0.1 Å, 0.5 Å and 1.0 Å of internal volume in MD melt structures of CA phases. Fractions sum to unity for zeo++ probe radius of 0.1 Å.

Partial atomic volume fractions plotted in Figure 3D scale almost linearly with x=CaO/(CaO+Al_2O_3) and demonstrate that the densities of the CaO and Al_2O_3 subcomponents are almost fixed in all CA cement phases. This is borne out by the Binary Model parameterised by the densities of CaO and Al_2O_3 liquids. Coupled with the O-speciation in the liquids being very similar to the corresponding crystals, supports the conclusion that nucleation of CA phases in cement formation must be facile while glass forming ability (GFA) is poor.

5. Supplementary Method 5. Oxygen and aluminum speciation in CA melts and crystals

Both oxygen and aluminum display a variety of configurations whose proportions change in CA melts between Al_2O_3 and CaO. Most of these configurations are also present in

the corresponding crystals, as demonstrated for oxygen in Figure 2D. The spatial random distribution of O(0), O(1), O(2) and O(3) configurations, together with Al^{IV}, Al^{V} and Al^{VI} polyhedra can be visualized in Figure 5A.

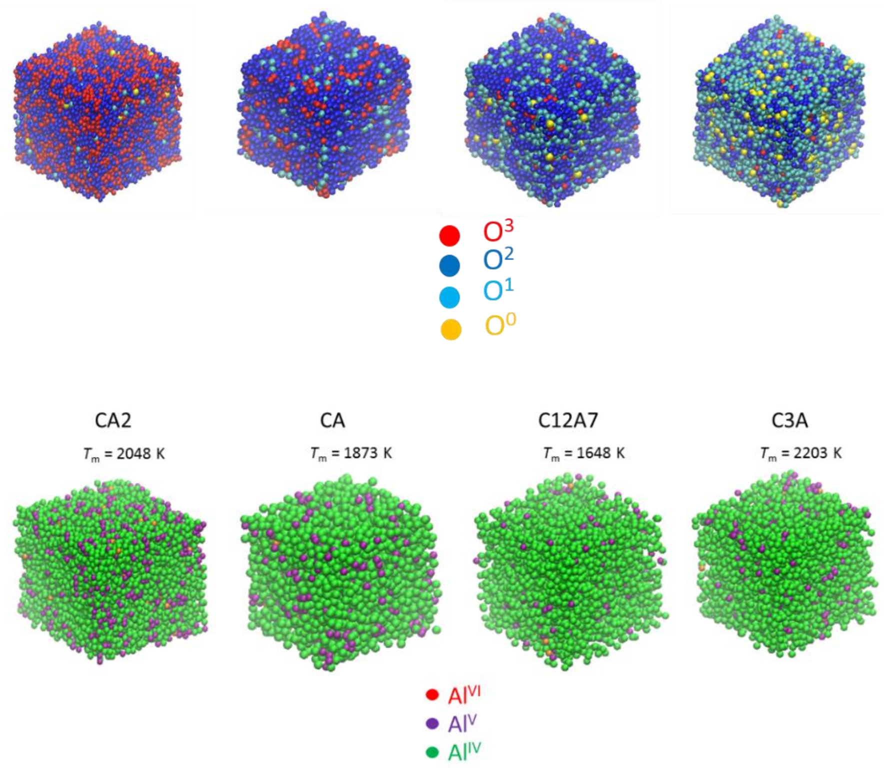

Figure S5A O^n configurations (Top) and Al^n polyhedra (Bottom) from MD simulations for CA liquids at their respective melting points T_m.

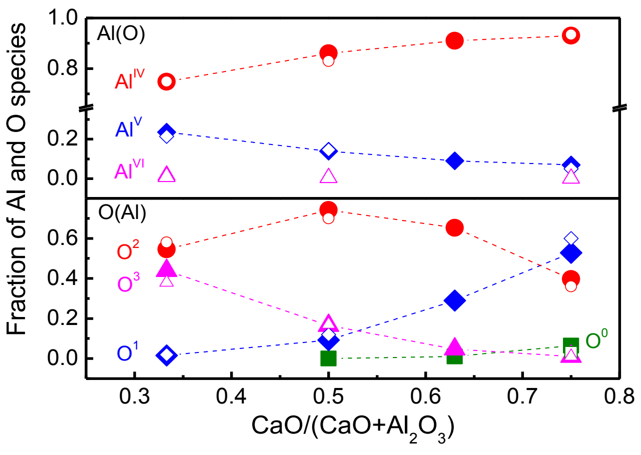

Figure S5B. Distributions of Al(O) species and O(Al) species with CA composition for MD simulated CA melts. Solid symbols: current MD simulations; Open symbols: results from previous MD simulations combined with neutron and x-ray diffraction measurements.[6,9,47]

There is broad agreement of Al-speciation in molten CA phases reported previously from ALF of Al-K from L-edge X-ray Absorption Spectroscopy[5] and ^{27}Al NMR[7] experiments. While Al is found to be mainly tetrahedral from CA2 to C3A melts as it is in crystalline phases, there is a significant fraction of Al^{V} polyhedra of between 22% and 16%.

O-speciation of melts follows crystalline CA phases (Figure 2D). Melt structures evolve from CA2 with its oxygen-deficient random network (ODRN), through the CRN corner-sharing tetrahedral network of CA, to C12A7, where the aluminate network starts to fragment, becoming even more incomplete for C3A which lies at the percolation threshold for Al_2O_3 (Figure 3D). Crystalline phases follow similar topological trends, changing from the oxygen-deficient CA2 grossite network of corner sharing AlO_4 tetrahedra combining bridging oxygens (O^2) with triclusters (O^3), through the charge-compensated β-tridymite continuous Q^4 tetrahedral network of CA where oxygens are chiefly bridging (O^2). This is followed by the complex but incomplete aluminate maynite network of C12A7, to C3A where Q^2 AlO_4 tetrahedra, each with two terminal oxygens (O^1) and two bridging oxygens (O^2), are restricted to a broken network of 6-fold rings. Throughout, Ca is octahedrally co-ordinated, but distorted 6- and 9-fold sites in CA to near perfect octahedral sites in C3A.

6. Supplementary Method 6. Steps in atomic volume and excess entropy at $T_{f\text{-}s}$.

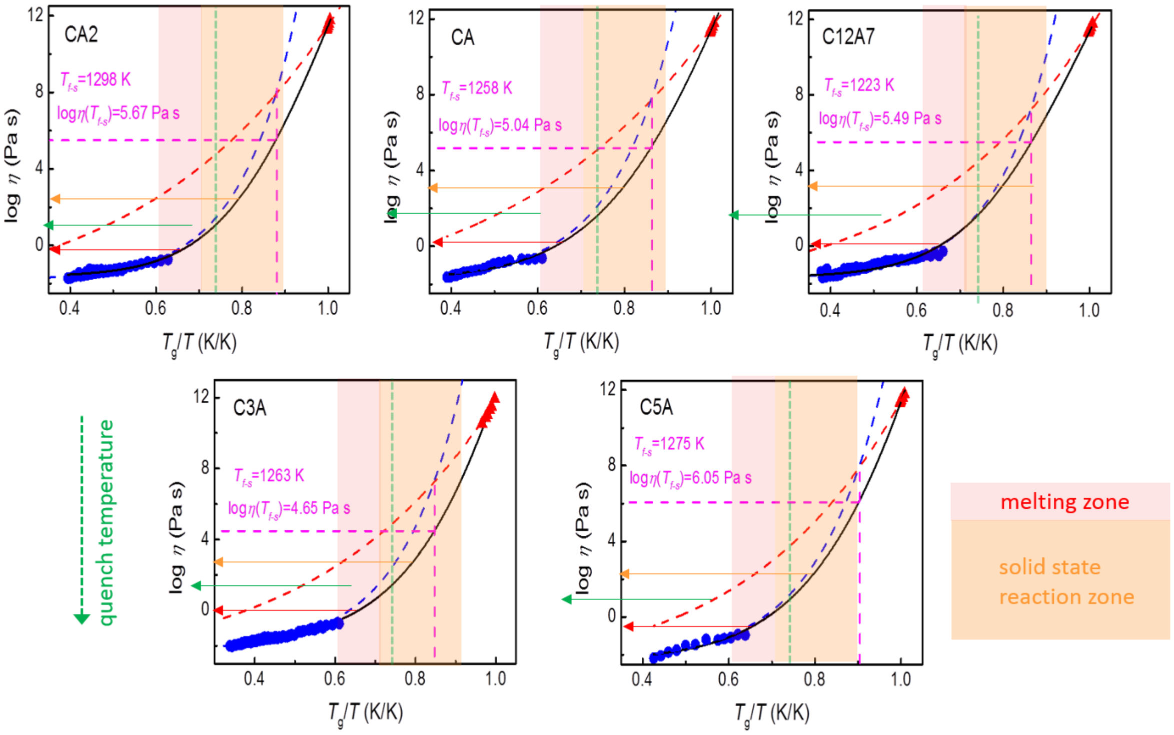

Figure S6A. *f-s* transitions identified across the calcium-aluminate liquids modelled using MYEGA analysis.[28] Cement processing zones are shown together with quenching temperature.

Figure 4A and Figure S6A present the viscosity of CaO-Al_2O_3 liquids near T_m and supercooled liquids near T_g. The curves were fitted using the MYEGA model[28] and are fully consistent with viscosity data in the two temperature regions, enabling *f-s* transitions to be indentified at ~1.2T_g (Figure 1C).

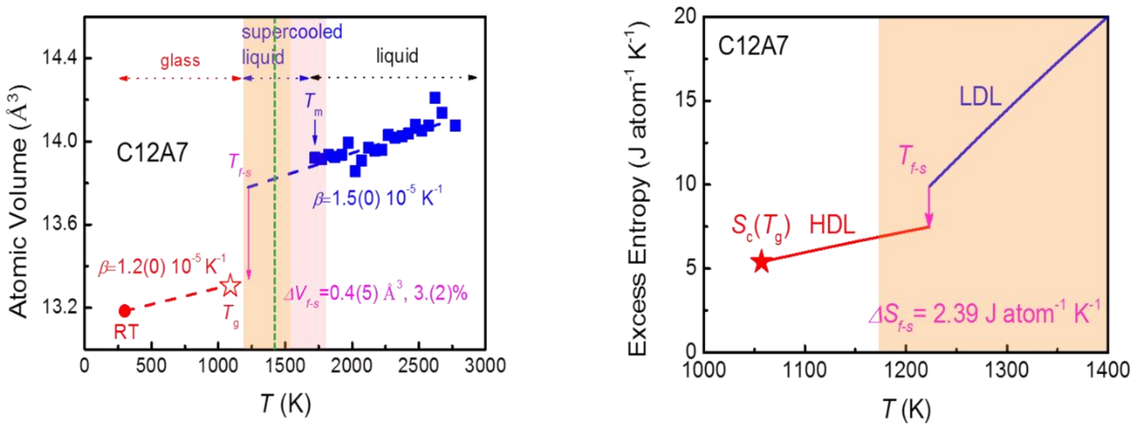

Figure S6B. *f-s* step in atomic volume (left) and excess entropy (right), modelled using ALF measured densities and CTEs, and Adam-Gibbs analysis of fragile and strong components, respectively. Cement processing zones are superimposed.

Figure S6B shows modelling of the step in atomic volume and excess entropy at the *f-s* temperature ($T_{f\text{-}s}$) for C12A7. The liquid atomic volume was calculated by using ALF measured liquid densities and CTEs (Figure S3), while the glass atomic volume was calculated by using measured glass density at RT and CTEs of crystals from refs. 51 and 53-56. The step in atomic volume ΔV occurs at $T_{\text{f-s}}$ where the mismatch in viscosity occurs (Figure S6A). The equivalent excess entropy step ΔS was determined by using Adam-Gibbs analysis of fragile and strong components of viscosity data.

7. Supplementary Method 7. Cement processing temperatures and viscosities.

Cement processing temperatures included in Figure 4 and Figure S6 are the standard multi-stage zones used in rotary kilns or vibratory furnaces[2,3] viz. the solid-state reaction (sintering) zone 1173 K-1573 K, the melting (clinkering) zone 1573 K-1773 K, and the quenching temperature T_Q 1089 K. The solid-state reaction zone lies above the glass transition temperatures T_g of all CA phases but incorporates the crystallization temperatures

T_p and *fragile-strong* transition temperatures $T_{f\text{-}s}$. The melting zone extends from the melting temperatures T_m of C12A7 and CA, confirming the melting of other phases results from eutectic processes. T_Q lies above $T_{f\text{-}s}$ in the ergodic region for the separate CA phases and, therefore, above the dynamic cross-over in the supercooled state.[23] This covers the region where the plateau in heterogeneous nucleation growth rates extends.[15] Quenching from this temperature will promote the crystallisation of highly labile cement phases like CA2, CA and C3A, leaving C12A7, which has relatively good GFA, the only likely vitreous phase (Figure 2B).

Finally, the viscosities of individual CA phases read directly from Figure 6A at the average sintering (1373 K), melting (1673 K) and quenching (1473 K) temperatures are plotted in Figure 4E and Figure S7, covering ~1000 Pa.s (Working Point),~1 Pa.s, (Melting Point) and ~20 Pa.s (Practical Melting Point) respectively. The various working and melting points are those recognised in Glass Technology and throughout industry.

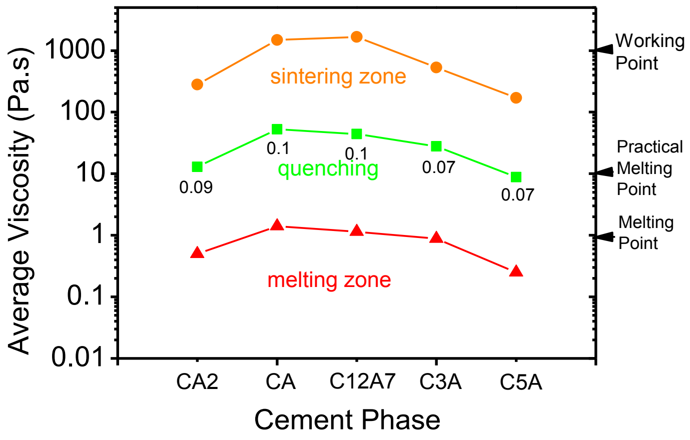

Figure S7. Average viscosities of individual cement phases for the sintering, melting and quenching zones in rotary kilns.[2,3] These are read from the total viscosity curves of CA phases presented in Figure S6A.

S10. References

51. A. S. Madhusudhan Rao, K. Narender, *J. Thermodyn.* **2014**, 2014, 123478

52. S. K. Srivastava, P. Sinha, M. Panwar, *Indian J. Pure Appl. Phys.* **2009**, 47, 175.

53. H. M. Lu, Q. Jiang, *phys. stat. sol (b)* **2004**, 241, 2472.

54. S. Jonas, F. Nadachowski, D. Szwagierczak, G. Wójcik, *J. Eur. Ceram. Soc.* **2006**, 26, 2273.

55. S. Jonas, F. Nadachowski, D. Szwagierczak, *Ceram. Int.* **1999**, 25, 77.

56. R. W. Whatmore, C. O'Hara, B. Cockayne, G. R. Jones, B. Lent, *Mater. Res. Bull.* **1979**, 14, 967.
